# Charting the Future of Canadian Adult Acute Myeloid Leukemia (AML) Laboratory Testing: A Canadian Leukemia Study Group Current State Mapping of Diagnostic AML Laboratory Practice

**DOI:** 10.3390/curroncol33090505

**Published:** 2026-08-25

**Authors:** Tina Yu Xuan Luo, Sila Usta, Eric McGinnis, Cheryl A. Mather, Julie Bergeron, Tanya Gillan, Etienne Mahe, José-Mario Capo-Chichi, Philip Berardi, Paul C. Park, Doha Itani, Ashish Rajput, Benjamin Chin-Yee, Fei-Yu Han, Darci T. Butcher, Jennifer Fesser, John DeCoteau, Graeme Quest, Elizabeth McCready, Hubert Tsui

**Affiliations:** 1 Biological Sciences Platform, Sunnybrook Research Institute, Toronto, ON M4N 3M5, Canada; 2 Division of Hematological Pathology, Department Laboratory Medicine and Molecular Diagnostics, Precision Diagnostics and Therapeutics Program, Sunnybrook Health Sciences Centre, Toronto, ON M4N 3M5, Canada; 3 Vancouver General Hospital, University of British Columbia, Vancouver, BC V5Z 1M9, Canada; 4 Molecular Pathology (North Sector), Alberta Precision Laboratories, Edmonton, AB T6G 2B7, Canada; 5 Department of Laboratory Medicine and Pathology, University of Alberta, Edmonton, AB T6G 1C9, Canada; 6 CEMTL Installation Maisonneuve-Rosemont, University of Montreal, Montreal, QC H1T 2M4, Canada; 7 The Canadian Leukemia Study Group/Groupe Canadiene D’étude sur la Leucémie (CLSG/GCEL), Toronto, ON M4N 1A7, Canada; 8 Department of Pathology Central Zone, Nova Scotia Health, Halifax, NS B3H 1V8, Canada; 9 Department of Pathology, Dalhousie University, Halifax, NS B3H 4R2, Canada; 10 Departments of Pathology & Laboratory Medicine, and Division of Hematology & Hematological Malignancies, Department of Medicine, Cumming School of Medicine, University of Calgary, Calgary, AB T2N 4N1, Canada; 11 Division of Hematology & Molecular Pathology Programme, Alberta Precision Laboratories, Calgary, AB T2N 2T9, Canada; 12 Department of Laboratory Medicine and Pathobiology, Temerty Faculty of Medicine, University of Toronto, Toronto, ON M5S 1A8, Canada; 13 Division of Clinical Laboratory Genetics, University Health Network, Toronto, ON M5G 2C4, Canada; 14 Department of Pathology and Laboratory Medicine, The Ottawa Hospital, University of Ottawa, Ottawa, ON K1H 8L6, Canada; 15 Department of Pathology, University of Manitoba, Winnipeg, MB R3E 3P5, Canada; 16 Diagnostic Services, Shared Health, Winnipeg, MB R3C 0C4, Canada; 17 Molecular Diagnostics and Cytogenetics, Saint John Regional Hospital, Horizon Health, Saint John, NB E2L 4L2, Canada; 18 Pathology and Laboratory Medicine, Newfoundland Health Services, St John’s, NL A1B 3V6, Canada; 19 Schulich School of Medicine & Dentistry, Western University, London, ON N6A 5C1, Canada; 20 Department of Pathology and Laboratory Medicine, and Division of Hematology, Department of Medicine, London Health Sciences Centre, London, ON N6A 5W9, Canada; 21 Laboratory Medicine, Memorial University, St. John’s, NL A1B 3V6, Canada; 22 Department of Pathology and Molecular Medicine, McMaster University, Hamilton, ON L8S 4K1, Canada; 23 Hamilton Regional Laboratory Medicine Program, Hamilton Health Sciences and St Joseph’s Healthcare, Hamilton, ON L8N 4A6, Canada; 24 Transfusion Medicine and Laboratory Hematology, Queen Elizabeth Hospital, Health PEI, Charlottetown, PE C1A 8T5, Canada; 25 Department of Pathology and Laboratory Medicine, College of Medicine, University of Saskatchewan, Saskatoon, SK S7N 5E5, Canada; 26 Department of Pathology and Molecular Medicine, Queen’s University, Kingston Health Sciences Centre, Kingston, ON K7L 3N6, Canada; 27 Department of Immunology, Temerty Faculty of Medicine, University of Toronto, Toronto, ON M5S 1A8, Canada

**Keywords:** acute myeloid leukemia, laboratory hematology, hematological pathology, biomarker, targeted therapy, quality improvement

## Abstract

Clinical decision making in Acute Myeloid Leukemia (AML) critically relies on rapid genomic characterization. To better understand the AML diagnostic landscape in Canada, the Canadian Leukemia Study Group (CLSG) conducted a survey of laboratory hematology leadership (n = 18) at 16 laboratories across 10 provinces, administered using Google Forms in September 2024. Nearly all surveyed sites were equipped to deliver a full suite of testing platforms through existing on-site infrastructure or laboratory partnerships. Reporting practices varied in terms of genomic integration into bone marrow results and the use of AML classification systems. Turn-around-time (TAT) targets were predominantly determined through internal institutional consensus (62%) or recommendations by provincial cancer agencies/international groups (44%). TAT reduction was a top priority for 56% of laboratories, suggesting timely biomarker results to be an active area for improvement. Various treatment-determining biomarkers were frequently assessed as rapid-tests (defined as a 5-day TAT), including *FLT3*-ITD (69%), *FLT3*-TKD (56%), and *NPM1* (56%), while others such as *IDH1* and *TP53* were rapid at a limited number of laboratories. Respondents demonstrated a strong shared interest in joint projects such as the validation of AML measurable residual disease (MRD) assays (56%). There was also unanimous support for establishing CLSG AML laboratory consensus guidelines. This survey documents the current state of Canadian AML laboratories and provides a foundation for future shared development projects.

## Simple Summary

Acute Myeloid Leukemia (AML) is an aggressive blood cancer requiring multiple types of laboratory tests for diagnosis, prognosis, and treatment decision making. To better prepare Canadian laboratories for the changing needs in AML patient care, we sought to capture the current state of AML diagnostic testing in Canada through a Canadian Leukemia Study Group (CLSG)-initiated survey directed towards key laboratory hematology stakeholders. We probed numerous themes, including testing methods, turn-around-times (TAT), algorithmic workflows, reporting preferences, and areas for future laboratory investment. Our findings show inter- and intra-provincial variation in AML laboratory practices, identifying opportunities for the CLSG to support harmonization and collaborative validation efforts. This survey, the first of its kind in Canada, fostered collective interest among acute leukemia laboratory leadership for formal shared advocacy and communication. The CLSG plans to leverage this sentiment as increasing demand for biomarker dependent therapies (e.g., AML measurable residual disease) necessitate laboratories work together to best deliver state of the art AML therapy to all Canadians.

## 1. Introduction

Clinical decision making in the management of Acute Myeloid Leukemia (AML) relies on rapid genomic characterization [1,2,3] independent of patient fitness for intensive or less-intensive therapies [1,4]. New and emerging targeted agents rely on genomic biomarkers [1,5], which impact upfront treatment as well as later in the patient journey where AML measurable residual disease (MRD) [6,7] is expected to influence allogeneic transplant decisions and the type/duration of maintenance treatment [3,8]. For laboratory specialists, another level of complexity is the co-existence of both the 2022 World Health Organization’s (WHO) classification system [8] and the 2022 International Consensus Classification (ICC) of myeloid neoplasms and acute leukemias [3].

While morphological and phenotypic assessment for AML diagnosis remains a mainstay, ancillary genomic tests are fundamental for classification and risk assessment [2]. For targeted treatments, genomic biomarkers also dictate eligibility and reimbursement. As a medical emergency [9], rapid genomic characterization of AML is thus of paramount importance [10,11].

In Canada, ensuring timely and equitable access to comprehensive laboratory testing fulfills a core tenet of universal healthcare. However, the status of genomic testing necessary for both current standard of care treatment and emerging therapies in adult AML in Canada has not been formally assessed. On behalf of the Canadian Leukemia Study Group (CLSG) [12], we sought to document the current national diagnostic landscape by conducting a survey of laboratory hematology leadership. To evaluate the readiness of Canadian hematology laboratories to meet evolving international AML diagnostic standards [3], we queried regional differences in methodology, turn-around-times (TAT), algorithmic workflows, and reporting preferences (e.g., WHO vs. ICC) and aimed to outline areas for future laboratory cooperation. Our findings provide the first systematic evaluation of laboratory AML diagnostic practices in Canada, establishing a foundation for future Canada-wide laboratory initiatives. This CLSG goal is also aligned with Canada’s Drug Agency, which is involving laboratories earlier in the regulatory approval pathway (e.g., Assessment Framework for Biomarkers Used in Cancer Care) to better align therapeutic recommendations with laboratory preparedness [13].

## 2. Materials and Methods

This survey was designed to address themes of laboratory leadership diversity, institutional reporting practices, AML genomics (rapid testing type and methodology), cytogenetics, Next Generation Sequencing (NGS), TAT, and priority areas for laboratory collaboration. Survey conception was triggered by evolving biomarker-dependent AML therapies requiring faster TAT [2,14,15]. With an aim to better inform the CLSG on laboratory readiness, we engaged a core group of 6 hematopathologists and genetic and genomic diagnostic (CCMG credentialled) scientists to comment on the survey framework initially drafted by TYXL/HT based on clinical practice experience. Following iterative improvements on survey clarity and debugging of questionnaire logic, the survey was unanimously approved by this initial core group for distribution. The final survey consisted of a mix of free-text, multiple choice, select-all, and Likert-scale items, totalling 64 questions across 13 subsections (File S1). The survey was administered using Google Forms in September 2024 and therefore reflects laboratory practices at the time of data collection. Hematology laboratory leadership (n = 18) from 16 laboratories across 10 Canadian provinces participated. Laboratory participants were identified through clinical CLSG board members, which represented all major acute leukemia treating centres in Canada. Where responses were unclear or discrepant, respondents were directly contacted by TYXL/SU/HT for clarification. 

The survey discussed the following molecular and cytogenetic targets: 

*NPM1* (Nucleophosmin 1); 

*FLT3* (FMS-like tyrosine kinase 3); 

*FLT3*-ITD (Internal Tandem Duplication);

*FLT3*-TKD (Tyrosine Kinase Domain); 

*IDH1* (Isocitrate Dehydrogenase 1); 

*IDH2* (Isocitrate Dehydrogenase 2); 

*TP53* (tumor protein p53); 

*MECOM* (MDS1 and EVI1 Complex Locus); 

*PDGFRA* (Platelet-Derived Growth Factor Receptor Alpha) rearrangements; 

*RUNX1::RUNX1T1*/t(8;21)(q22;q22.1) (Runt-related transcription factor 1:: *RUNX1* Partner Transcriptional Co-Repressor 1);

*CBFB::MYH11*/inv(16)(p13.1q22) or t(16;16)(p13.1;q22) (core binding factor beta:: myosin heavy chain 11); 

*PML::RARA*/t(15;17)(q24.1;q21.2) (Promyelocytic Leukemia:: Retinoic Acid Receptor Alpha); 

*BCR::ABL1*/t(9;22)(q34.1;q11.2) (Breakpoint cluster region::Abelson murine leukemia 1); 

*KMT2A* (Lysine Methyltransferase 2A) rearrangements.

## 3. Results

### 3.1. Survey Participation and Respondent Characteristics

**Figure 1:**
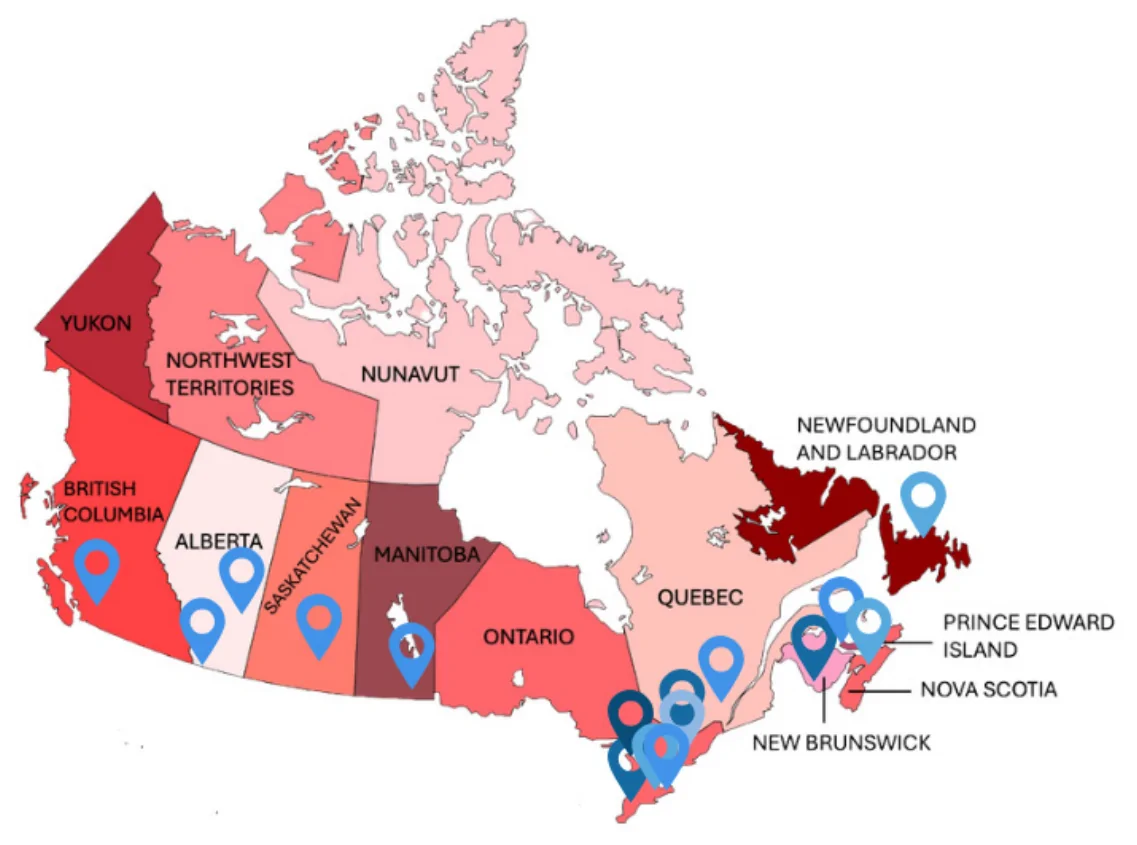
Geographical spread of surveyed laboratories across Canada (n = 10 provinces). Created with mapchart.net. Creative Commons Attribution-ShareAlike 4.0 International License (CC BY-SA 4.0).

Eighteen hematology genomic laboratory leaders from 16 institutions across 10 Canadian provinces completed the survey, Table 1. The geographical distribution is shown in Figure 1. To probe the variety of professional backgrounds in molecular laboratory hematology leadership, we asked participants for their medical/scientific credentials. Respondents included MDs with specialized training in hematological pathology (50%, 9/18), diagnostic and molecular pathology (formerly anatomic pathology) (17%, 3/18), internal medicine (11%, 2/18), and diagnostic and clinical pathology (formerly general pathology) (6%, 1/18) with over a quarter of respondents having a PhD (28%, 5/18) and several with fellowship training in molecular genetics/cytogenetics/genomics (MGCG) (22%, 4/18) and molecular pathology (MP) (22%, 4/18), Table 1. 

Participating institutions represented all major provincial acute leukemia hubs. Canada’s most populous province, Ontario, was well represented with seven institutions (44% of total). Other centres included British Columbia (Vancouver General Hospital/British Columbia Cancer Agency), Alberta (Alberta Precision Laboratories, Edmonton and Calgary), Saskatchewan (University of Saskatchewan), Manitoba (Shared Health Manitoba), Quebec (Hôpital Maisonneuve-Rosemont), Newfoundland (Newfoundland Health Services), New Brunswick (Saint John Regional Hospital), Nova Scotia (Nova Scotia Health), and Prince Edward Island (Health PEI), Table 1. 

### 3.2. On-Site Testing Capabilities

**Figure 2:**
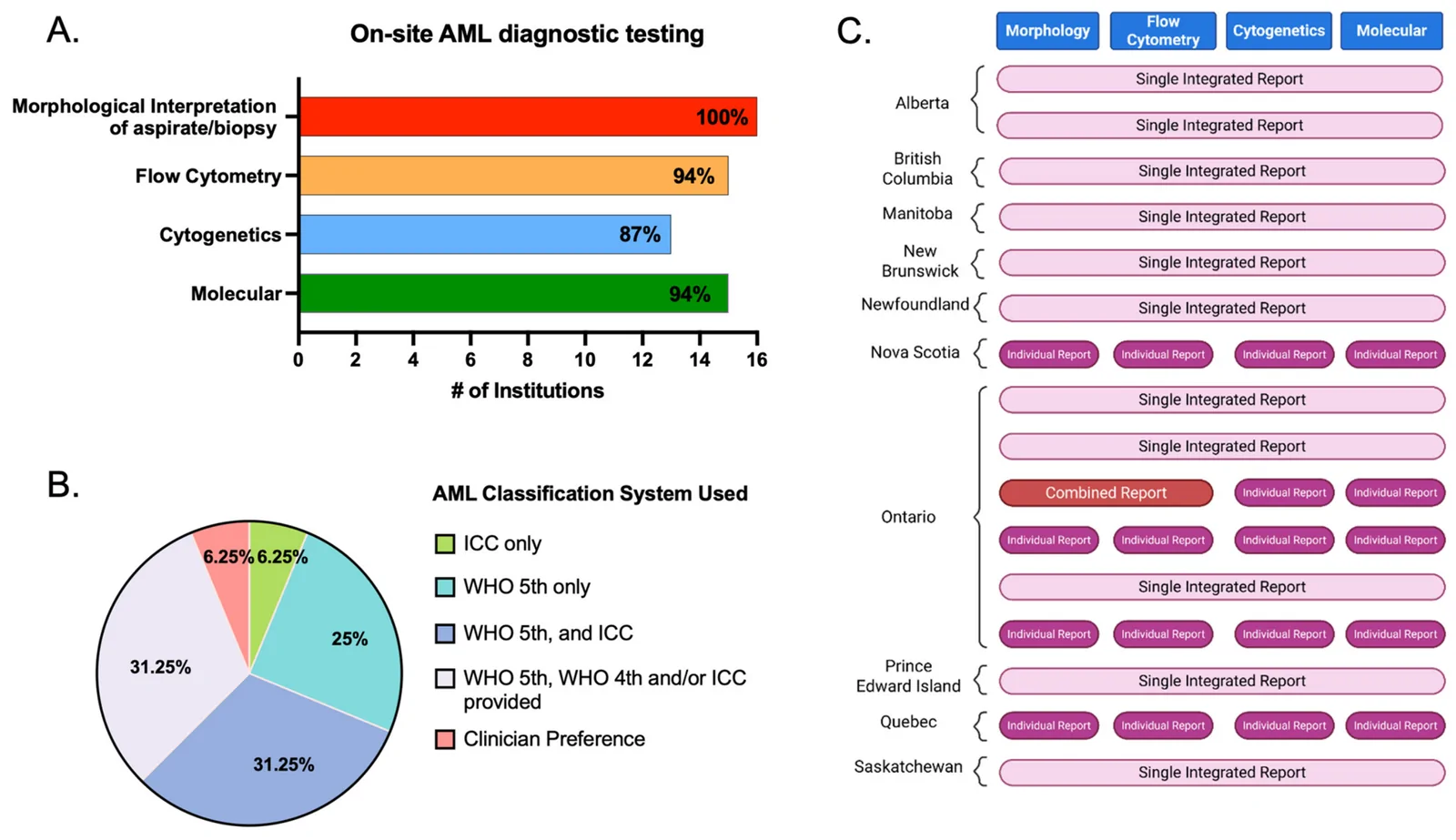
Institutional bone marrow reporting practices. (**A**) Availability of on-site diagnostic testing methodologies at surveyed sites. (**B**) AML classification systems employed. (**C**) Delivery and integration of diagnostic testing results. Created in BioRender. Usta, S. (2026) https://BioRender.com/4hm5zsp, (accessed on 7 July 2026).

Given that acute leukemia diagnosis and prognostication involve the integration of morphology, flow cytometry, and genomic investigations, we asked surveyed sites for physical on-premise capabilities to provide the entire suite of acute leukemia diagnostics. Primary gold-standard morphological assessment of an aspirate and biopsy was performed at 100% of sites surveyed (16/16 sites), and nearly every site offered at least flow cytometry (94%, 15/16), molecular testing (94%, 15/16), and slightly less frequently, cytogenetics (87%, 13/16 sites) (Figure 2A).

### 3.3. Institutional Practices for AML Classification and Integrated Reporting 

Currently, two classifications systems are used for the diagnosis of AML, the ICC [3] and WHO Classification of Tumors—Hematolymphoid (5th edition) [8]. AML classification in Canada showed mixed adoption, with 25% (6/16) of respondents using the WHO 5th edition only, 31% (5/16) using both WHO 5th edition and International Consensus Classification (ICC) (2022), and 31% (5/16) using both WHO 4th and 5th editions and/or ICC, with a minority (1/16) using ICC only (Figure 2B). 

Integration of molecular/cytogenetic testing results into a single comprehensive bone marrow report was common practice at most sites (69%, 11/16), while the remaining sites (31%, 5/16) issued standalone or semi-integrated reports for these results (Figure 2C). 

### 3.4. Turn-Around-Time (TAT) Benchmarks

**Figure 3:**
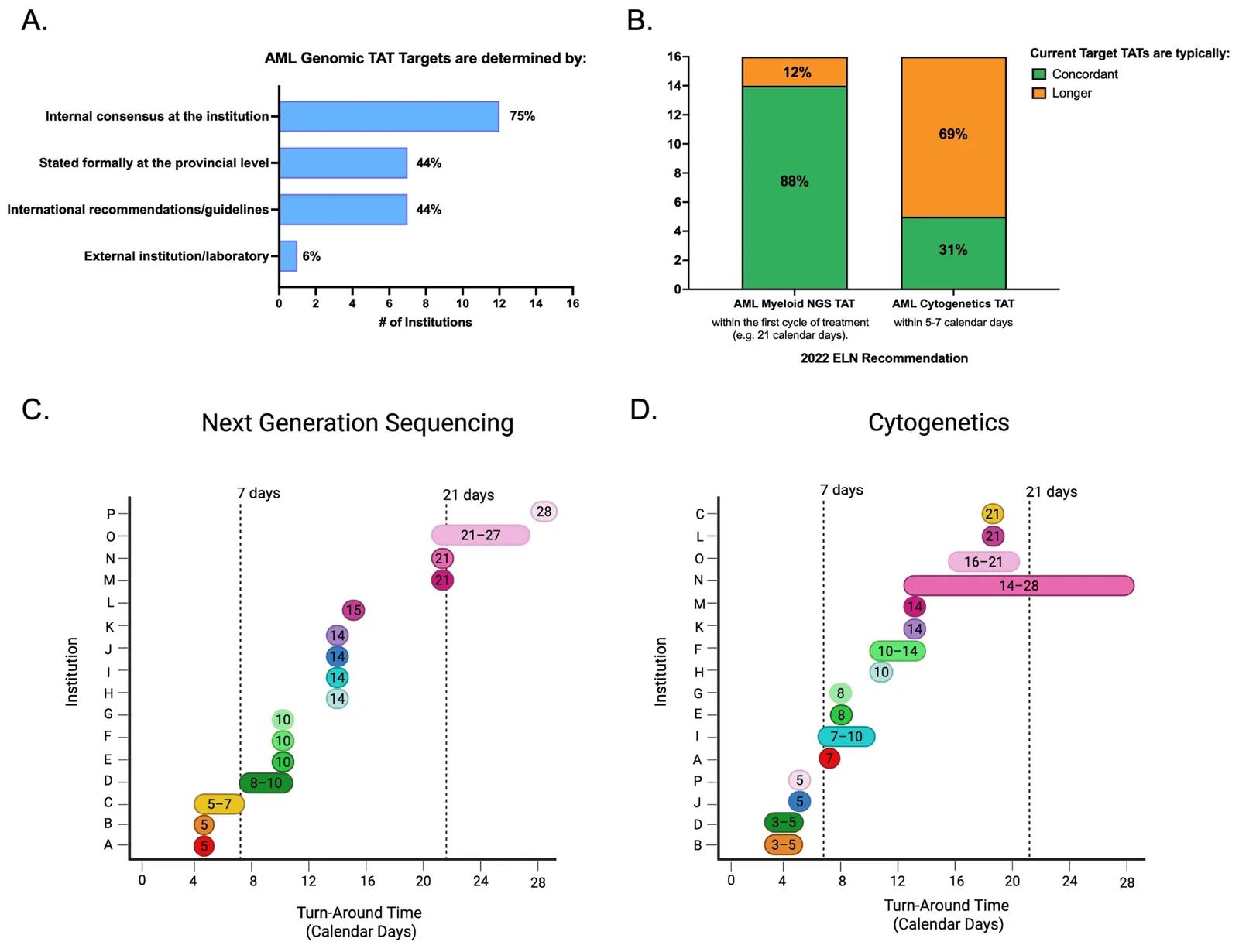
Turn-around time benchmarks. (**A**) Sources of TAT guidelines. (**B**) The proportion of institutions aligned with target TATs outlined in the 2022 European Leukemia Network recommendations. (**C**,**D**): Created in BioRender. Usta, S. (2026) https://BioRender.com/jpfkzer, accessed on 7 July 2026. Institution-specific NGS and cytogenetic target TATs. (Individual institutions are colour-coded, alphabetically designated and ordered by AML NGS TAT (**C**) and re-ordered by Cytogenetic TAT (**D**)).

Expedited laboratory testing for AML is important for upfront clinical decision making, and therefore, TAT targets are generally shorter for suspected AML presentations compared to chronic myeloid neoplasms such as myelodysplastic syndrome (MDS) and myeloproliferative neoplasm (MPN). Survey results showed that TAT is an institution-specific management decision, with most institutions (75%, 12/16) generating internal guidelines, 44% (7/16) referencing provincial or international recommendations, and 6% (1/16) relying on external institutions (Figure 3A). 

International guidelines such as the 2022 European Leukemia Network (ELN)-recommended TATs for AML diagnostic testing [2,16] segregate biomarkers into two main categories with more rapid biomarkers in 5–7 days (e.g., *NPM1*, *FLT3, IDH1,* and cytogenetics) and others within the first cycle of treatment. When assessing targeted panel myeloid NGS, most laboratories (88%, 14/16) reported meeting the recommended TAT of ≤first cycle or 21 calendar days, while only 31% (5/16) met the cytogenetics target of 5–7 calendar days (Figure 3B). 

A more detailed assessment of institution-specific myeloid NGS TAT targets ranged from 5 to 28 calendar days, with three distinct groups emerging: *1.* sites achieving rapid TATs within 5–7 days (19%, 3/16), *2.* sites achieving TAT within the ELN-recommended window (56% 9/16), and *3.* sites exceeding the 21-day target (25%, 4/16) (Figure 3C). 

In contrast to molecular TAT, cytogenetic TAT targets revealed challenges conforming to ELN benchmarks given that only a minority achieved a recommended 5–7-day TAT (n = 5), while most experienced longer TATs than ELN recommendations (Figure 3D), with a national mean of 11 days (n = 16). 

### 3.5. On-Site Rapid Testing Capabilities and Methodology 

**Figure 4:**
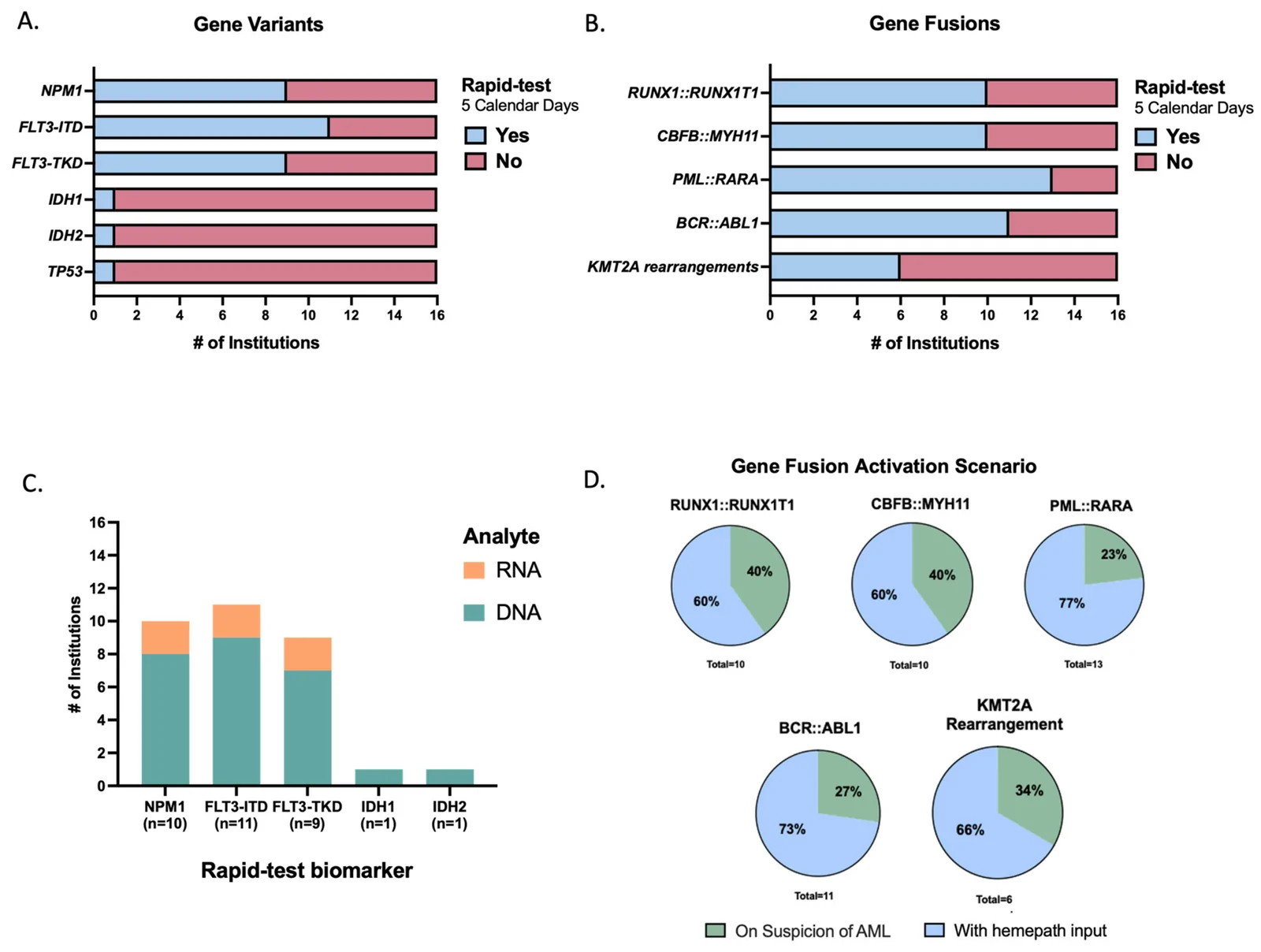
On-site AML rapid test capabilities. Availability of in-house rapid tests (defined as 5 calendar days) for gene variants (**A**) and gene fusions (**B**). (**C**) Analyte use per rapidly tested gene (RNA vs. DNA-based). (**D**) Gene fusion rapid testing activation protocol.

Certain AML biomarkers such as *PML::RARA*, *FLT3*-ITD, *FLT3*-TKD, and core binding factor (CBF) mutated leukemias’ influence-specific upfront therapy [17]. As such, the capabilities of surveyed laboratories to perform on-site rapid testing defined as five-calendar-day TAT was assessed. Several sites delivered rapid *NPM1* (56%, 9/16), *FLT3*-ITD (69%, 11/16), and *FLT3*-TKD (56%, 9/16), while only one site delivered rapid testing for *IDH1*, *IDH2*, and *TP53* at the time of the survey (Figure 4A). Overall, most laboratories offered rapid fusion testing for *RUNX1::RUNX1T1* (63%, 10/16), *CBFB::MYH11* (63%, 10/16), *PML::RARA* (81%, 13/16), and *BCR::ABL1* (69%, 11/16) but less often for *KMT2A* rearrangements (38% 6/16). Given rapid testing for AML involves the detection of a variety of genetic changes such as insertions, deletions, substitutions, and fusions, we probed the molecular analyte used for performing five rapid AML tests (Figure 4C). DNA is the preferred analyte for rapid testing of *NPM1, FLT3*-ITD, and *FLT3*-TKD with a minority of sites utilizing RNA-based assays (Figure 4C). For sites reporting rapid NPM1, FLT3-TKD/ITD, IDH1/2, and TP53, one laboratory reported the capability to detect these biomarkers using NGS within a 5-day TAT threshold, with the remainder using PCR/RT-PCR-based platforms.

From a genomic utilization standpoint, we next assessed the relevance of morphology and flow cytometry phenotype in triggering specific treatment-altering AML rapid fusion testing, such as CBF mutated leukemia and acute promyelocytic leukemia (APL). Most centres relied on hematopathology triage/review (60–77%) more often than automatic rapid test suite on suspicion of acute leukemia (23–40%) (Figure 4D).

### 3.6. AML Genomic Methodologies

**Figure 5:**
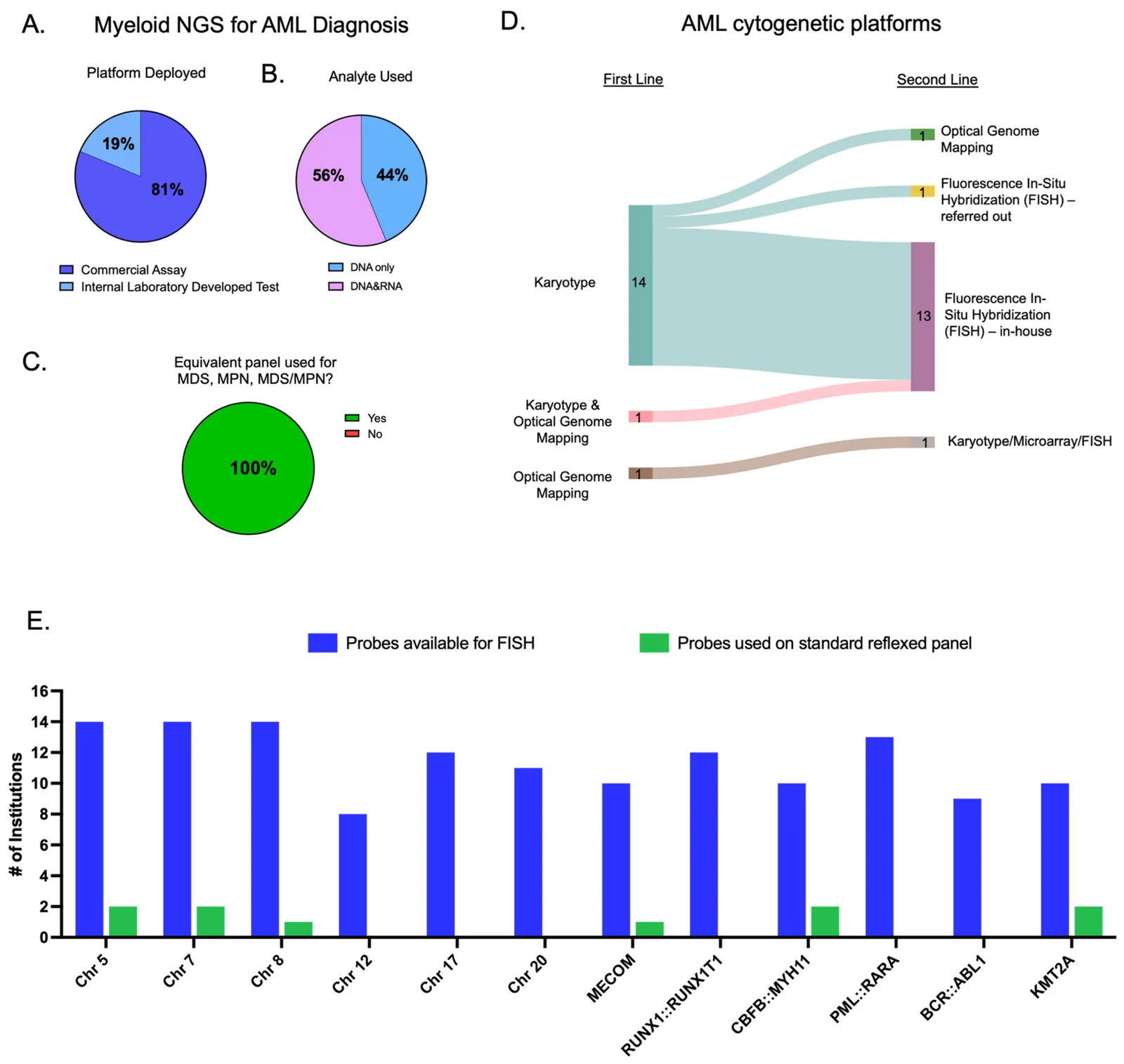
AML molecular and cytogenetic testing. (**A**–**C**) Myeloid NGS deployment. (**A**) Assay type. (**B**) Analyte use. (**C**) Similar NGS platform is also used for MDS, MPN, and MDS/MPN. (**D**,**E**) AML cytogenetics: (**D**) Overview of first and second-line cytogenetics methodology. (**E**) FISH probe availability across sites, including probes performed reflexively and by-request.

For AML molecular testing, deployment of commercial myeloid NGS panels was more common (13/16 81%) than internal laboratory developed tests (LDT) (3/16, 19%) (Figure 5A). DNA is the preferred analyte with many laboratories also utilizing RNA for fusion detection. Universally, broad-based myeloid neoplasm-targeted gene panels were used for AML as well as the assessment of myelodysplastic syndrome (MDS), myeloproliferative neoplasms (MPN), and MDS/MPNs. 

Cytogenetic methodology for AML was largely dominated by karyotype and fluorescence in situ hybridization (FISH) as first and second line, respectively (Figure 5D). Emerging technology such as Optical Genome Mapping (OGM) was deployed as first line in two (2/16, 12.5%) institutions and second line at one (1/16, 6.25%) institution (Figure 5D). FISH may be used as a rapid test for fusion detection but also in situations of karyotype failure. To determine if FISH is universally employed to salvage failed or suboptimal karyotypes, we assessed the presence of standardized probe panels for reflexed-FISH, with most respondents indicating that this was not the case (12/16, 75%). Indeed, while most laboratories had several probes available for FISH, these were mostly utilized on a per-request basis. Only some sites deploy automatically reflexed probes for chromosomes 5, 7, and 8 as well as *MECOM*-rearrangements and *CBFB::MYH11* and *KMT2A*-rearrangements upon failure of the first-line cytogenetic methodology (Figure 5E). 

### 3.7. Cryptic Rearrangements

**Figure 6:**
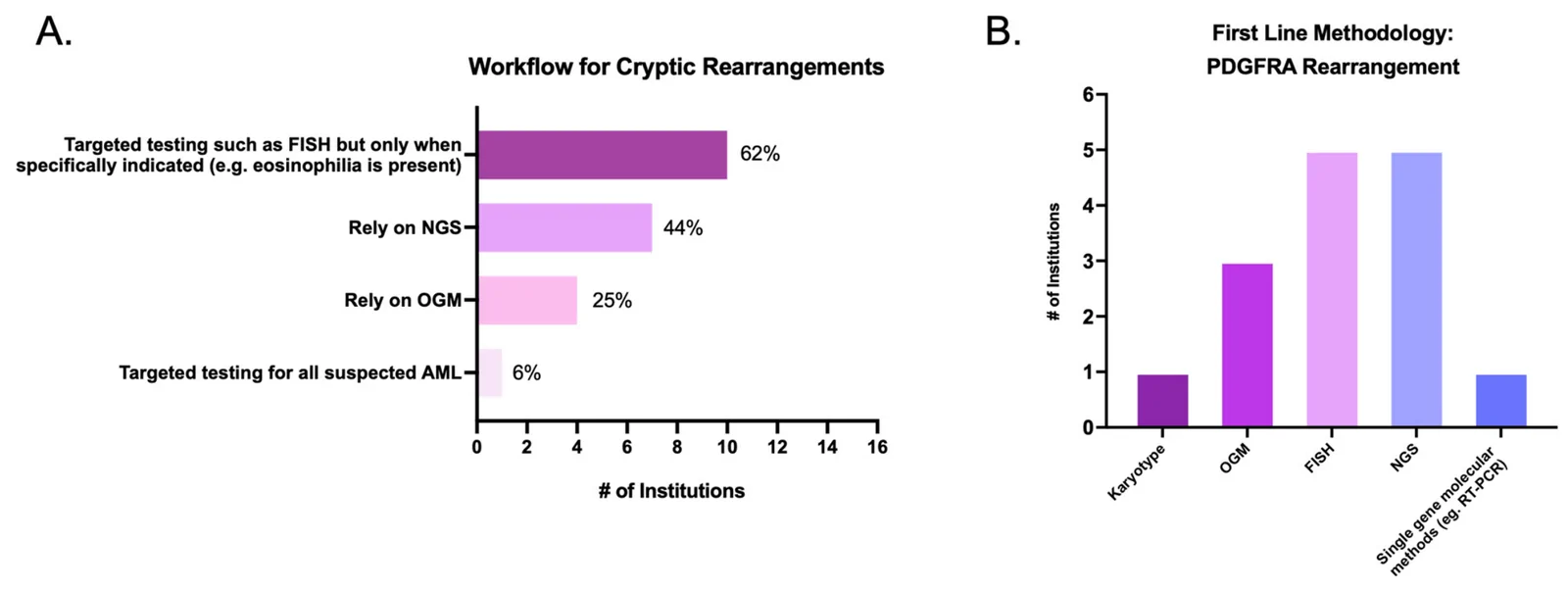
Strategy for AML with cryptic rearrangement. (**A**) Workflow deployed for detecting cryptic rearrangements. (**B**) PDGFRA rearrangement detection platform use.

As stated in the 2022 ICC and WHO [3,8], multiple recurrent and other rarer translocations are important for the prognosis and targeted therapy of AML [18]. Whereas karyotyping is the main modality for identifying structural variations (SV), there are clinically relevant fusions and translocations known to be cryptic when G-banding. When assessing alternate methodologies for identifying cryptic SVs; a variety of techniques are employed, with the most common techniques including FISH, NGS, and OGM. For most laboratories (Figure 6A), targeted FISH testing is triggered specifically on suspicion (e.g., by morphology or phenotype), whereas morphology- and phenotype-agnostic detection is provided by NGS and OGM. A minority of laboratories deployed targeted FISH for all suspected AML regardless of ancillary suspicion. To more directly explore a specific example of clinical relevance, we asked what first-line methodology was used when querying blast excess with eosinophilia, for example, *PDGFRA* rearrangements. As seen in Figure 6B, in this clinical scenario, FISH and NGS were the most frequent techniques, followed by OGM. 

### 3.8. Future Priority Projects

**Figure 7:**
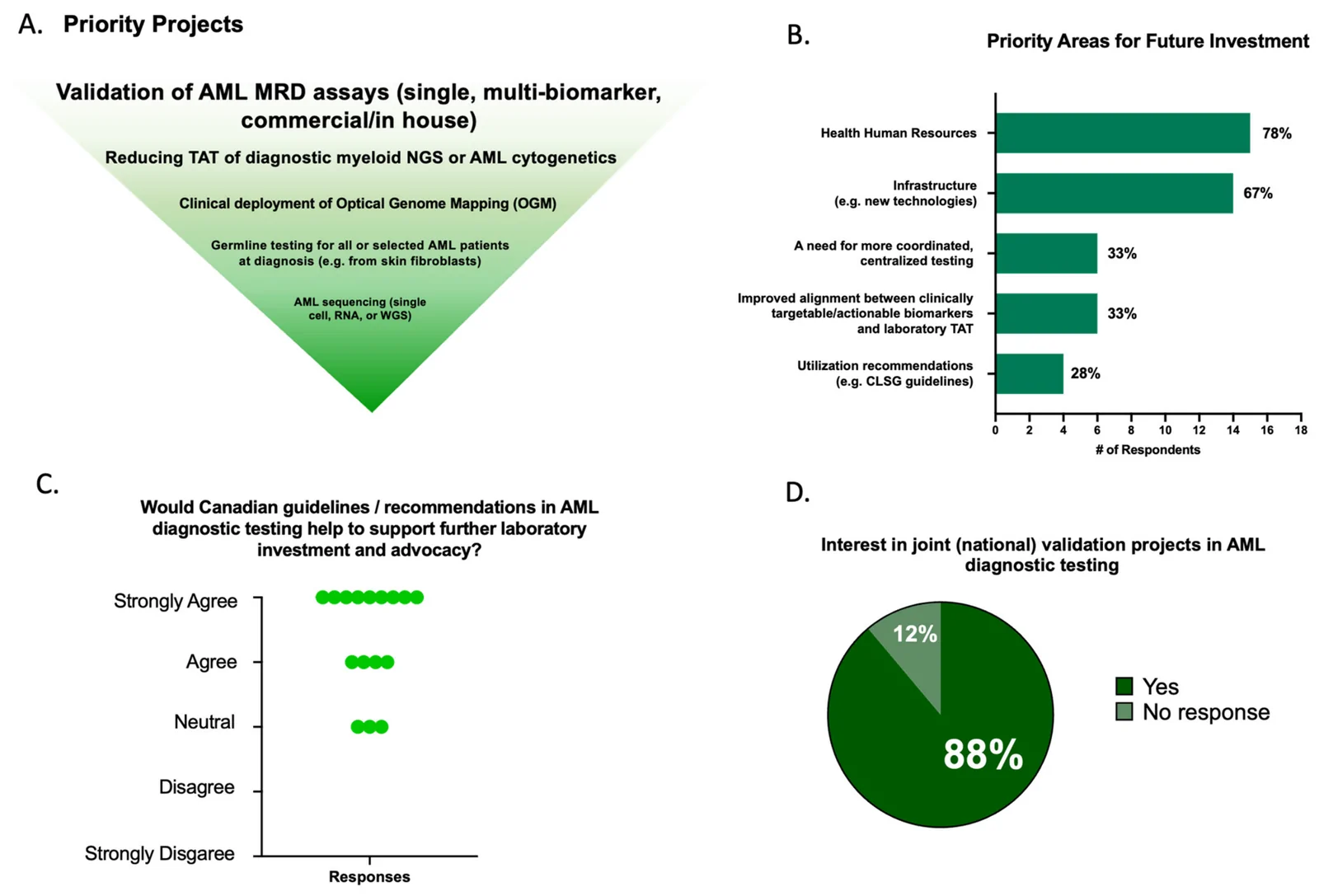
Future directions for AML diagnostic testing. (**A**) Priority projects ordered by frequency of selection by survey respondents. (**B**) Leading areas requiring investment. (**C**) Interest in harmonized nation-wide guidelines. (**D**) Interest in nation-wide validation efforts for AML diagnostic testing.

Priority projects are summarized in Figure 7. In a ranked list, respondents most frequently identified validation of AML measurable residual disease (MRD) assays as the leading priority, followed by improving TAT for diagnostic AML/myeloid NGS, AML cytogenetics, and clinical implementation of OGM [19]. Germline testing for hereditary hematological malignancies and other advanced AML sequencing approaches such as single-cell-based, transcriptomic, or whole-genome sequencing were also cited (Figure 7A). When asked about broader areas requiring future investment, the most selected domains were an increase in health human resources (14/16, 78%) and infrastructure, including new technologies (12/18, 67%) (Figure 7B). Finally, respondents expressed strong support for Canadian guidelines or recommendations for AML diagnostic testing as a mechanism to support laboratory investment and advocacy (Figure 7C). Broad interest in joint national validation projects was high, with (16/18, 88%) of respondents indicating interest to participate (Figure 7D).

## 4. Discussion

Genomic information plays an increasingly important role in the diagnosis, prognosis, and management of AML. In contrast to historic morphology-driven AML diagnosis and classification, current genomic diagnostic criteria allow for the diagnosis of AML even in the absence of increased blasts [3]. The CLSG aims to deliver world-class leukemia care to all Canadians. Given the dependency on laboratory biomarkers, we sought to assess the current state of AML diagnostic testing in Canada by directly contacting laboratory leadership at acute leukemia treatment centres in every Canadian province. 

Given the wide variety of training backgrounds, national advocacy in AML-associated genomic testing needs to be directed towards a diverse professional audience. Molecular pathology is practiced by different subspecialities such as anatomic pathology (now known as diagnostic and molecular pathology in Canada) and hematopathology, but directorship also relies on molecular scientists with PhD/CCMG training as well as clinical hematologists in provinces like Quebec. Population/geographic distribution is the main determinant of whether AML diagnostic testing is already ‘centralized’. For example, in British Columbia, Alberta, Saskatchewan, Manitoba, and the Atlantic region (Prince Edward Island, New Brunswick, Nova Scotia, and Newfoundland and Labrador), there is only one or two acute leukemia molecular diagnostic laboratories responsible for providing service to the entire province/region. In contrast, Ontario, with a large but geographically dispersed population, is serviced by multiple AML diagnostic laboratories, including several in Toronto and its surrounding area (UHN, Sunnybrook Health Sciences Centre, and Juravinski/Hamilton Health Sciences). As revealed in our survey data, differences in AML test methodology, activation procedures, and TAT exist both across provinces and within provinces. 

To our knowledge, this is the first systematic report to detail AML testing laboratories across Canada. Our survey results reveal differences in the AML classification system used, reporting formats, and TAT for rapid AML tests, cytogenetics, and myeloid NGS. International guidelines, such as the ELN for AML biomarker TAT, have been published [2], but for some jurisdictions these serve as aspirational targets. The identification of a wide range of TATs suggests that laboratories already meeting or outperforming ELN TAT criteria may be able to offer valuable operational insights to assist laboratories where achieving optimal TAT remains challenging. Given inter- and intra-provincial differences in testing, methodologies, and reporting, biomarker results may not be directly comparable between laboratories. The lack of methodological harmonization is compounded by two classification systems (WHO and ICC), for which the WHO 5th edition predominated overall with approximately 94% usage. Interestingly, the older WHO 4th ed. revised [20] was still used in almost a third of laboratories, possibly due to auditable terminology related to provincial drug reimbursement. Integrated bone marrow reporting that includes the incorporation of genomic results was cited by 69% of survey respondents. The time from genomic resulting to finalized ICC or WHO AML subclassification, however, was not directly surveyed. This potential lag may lead to interpretative discordances or ambiguity between AML diagnosticians and treaters and would be compelling to further explore. Here, compulsory synoptic reporting and use of laboratory information systems to monitor overdue/pending classification addendums may be of value. This survey also did not address potential genetic findings suggestive of hereditary predisposition/germline origin that impact AML classification, as this is the topic of a separate CLSG working group and guideline. 

In the category of biomarkers that impact upfront decision making, we found *NPM1* and *FLT3*-ITD/TKD TAT to be “rapid” when defined as target TAT of five calendar days (Figure 4A). This was an arbitrary threshold [2], acknowledging that provincial cancer agency TAT targets may differ and are also updated periodically (for example, see 2026 Ontario Health—Acute Leukemia Consensus Pathology Recommendations for Diagnosis and Monitoring, previously published in 2016 [21]). Other biomarkers, such as *IDH1/2, TP53* (Figure 4A) or *KMT2A* rearrangements (Figure 4B), were not tested in a rapid manner, suggesting laboratory factors and regulatory approval/funding for biomarker-targeted therapies were still undetermined or remained exploratory [13]. Of note, since the commissioning of this survey, molecular labs have strived to reduce TAT by using commercial targeted myeloid or pan-heme malignancy NGS platforms, including strategic prioritization of AML cases from other chronic myeloid specimens (MDS, MPN, MDS/MPN). This modification has helped to reduce TAT of rapid biomarkers such as *IDH1.* Further adoption of NGS panel-based approaches, including long read sequencing, may mitigate the need for additional standalone tests (i.e., *TP53*, *KMT2A*r). Detection of longer *FLT3*-ITDs at diagnosis, however, may still require separate methodology, as reviewed by the CLSG [22].

As a potential strategy to manage the supply–demand imbalance for rapid AML biomarker testing, we probed whether clinical triaging of suspected acute leukemia cases was common practice. Given a range of diagnostic ambiguity at bedside bone marrow procurement, laboratory physicians are positioned to triage acute leukemia test orders using combinations of morphology, flow cytometry, and clinical context. Indeed, this practice model was in use by up to 77% of reporting sites when asked if key gene fusion testing was performed for all suspected AML or on clinical/laboratory physician suspicion (Figure 4D). This data is the first to document the prevalence of hematopathology triage in AML-rapid test-related genomic utilization and may inform future hematology molecular practice, complementing “Choosing Wisely” initiatives [23]. A vast majority of laboratory stakeholders (67% of respondents) identified investment in molecular infrastructure as paramount (Figure 7B) with a specific theme of validating AML-MRD assays. This latter topic is timely given recent international guidance on AML-MRD from the ELN [2,6] and specifically on the technical aspects of *FLT3*-ITD MRD [24], for which the CLSG is actively promoting laboratory preparedness though a hub-based reference network.

While 94% of laboratories confirmed the availability of on-site flow cytometry (Figure 2A), we acknowledge that flow cytometry-based AML-MRD was not directly assessed in this survey. There is an unmet need to develop Canadian consensus practice guidelines for AML flow MRD where technical harmonization efforts have lagged efforts in the molecular realm. Diagnostic AML flow cytometry and AML flow MRD also differ greatly in infrastructure requirements and human resources given the complexity of interpretation, which necessitates additional subspecialty expertise and workload capture. Importantly, AML flow MRD is recognized as a valid modality by recent ELN-DAVID AML MRD recommendations [6]. Given localized Canadian experience in AML flow MRD, this topic is ideal for future CLSG advocacy and leadership [25].

To further highlight regional disparities in diagnostic AML testing, we queried the methodology in use for AML cytogenetics, including the specific scenario of accompanying eosinophilia [26]. At the time of the survey, a minority of laboratories (19%, 3/16 institutions; Figure 5D) had deployed genomic mapping technology for AML. Given the documented value for OGM in primary analysis of AML structural variations [19,27], our survey further reveals the different laboratory approaches to detecting often cryptic actionable fusions such as *PDGRFA* rearrangements (Figure 6B). Clinical implementation of genomic mapping technologies (or other long read sequencing methods) was selected as a priority project by 8/16 (50%) laboratories, suggesting this area should be emphasized for future CLSG-coordinated advocacy and collaboration [28]. Indeed, national guideline development was strongly supported by most respondents (Figure 7C), including robust support (88%) for Canada-wide joint validation projects.

Practical, real-world impacts of this national CLSG survey include establishing direct lines of communication with acute leukemia laboratory stakeholders across Canada. Prior to 2025, the CLSG had not included a primary laboratory specialist on its board of directors. This survey represents formal advocacy by the CLSG in molecular hematology and has directly led to a CLSG sponsored project for validation of high-sensitivity PCR-NGS for FLT3-ITD MRD [24], acting as a Canadian reference site for other laboratories. Importantly, these pre-emptive laboratory studies are coordinated with the approval of paradigm-changing treatment options, such as Health Canada regulatory recommendations for the use of FLT3 inhibitors in the peri-allogeneic transplant setting [17,29]. The importance of timely and harmonized laboratory biomarker deployment is reflected by contemporary technical guidance from ELN-DAVID [24]. Other direct outcomes of this survey are captured in upcoming national advisory boards, CLSG webinars, and industry collaborations for which both clinical and laboratory specialists have been invited to better bridge biomarker-driven developments in AML care.

Lastly, a principal tenet of publicly funded Canadian healthcare is universal access. Our survey has highlighted regional laboratory disparities that require ongoing monitoring and solutions, which will be highlighted for provincial cancer agencies as well as at national, CLSG supported conferences like the Canadian Conference on Myeloid Disorders (Ottawa, September 2027), where we have planned the agenda to specifically incorporate shared clinical and laboratory challenges. 

## 5. Conclusions 

In summary, the demand for genomic services and laboratory infrastructure supporting AML care is expanding. As technology improves and new biomarkers emerge, laboratories must be positioned to rapidly adapt to ensure high-quality, modern clinical care. Proactive communication with laboratory stakeholders earlier in the drug approval/reimbursement pathway is being incorporated by Canada’s Drug Agency and facilitation with national advocacy groups such as the CLSG will provide the practical laboratory frameworks necessary to deliver the most appropriate, biomarker-driven personalized therapy for Canadian AML patients. Our findings capture the importance of laboratory stakeholders in the evolving treatment of AML. 

## Figures and Tables

**Table 1:** Survey respondents.

Credentials	N=
PhD	5
MD-IM *	2
MD-HP *	9
MD-DMP *	3
MD-DCP *	1
Fellowship-MGCG **	5
Fellowship-MP **	4
**Respondents’ Institutions**	
Alberta Precision Laboratories—Edmonton	
Alberta Precision Laboratories—Calgary	
Shared Health Manitoba	
Saint John Regional Hospital	
Newfoundland Health Services	
Nova Scotia Health	
Kingston Health Sciences Centre	
University Health Network	
Sunnybrook Health Sciences Centre	
London Health Sciences Centre	
Hamilton Health Sciences	
The Ottawa Hospital	
Queen Elizabeth Hospital, Health PEI	
Hôpital Maisonneuve-Rosemont (HMR)	
University of Saskatchewan	

* Speciality training IM = Internal Medicine; HP = Hematological Pathology; DMP = Diagnostic and Molecular Pathology (formerly Anatomic Pathology); DCP = Diagnostic and Clinical Pathology (formerly General Pathology); ** Fellowship MGCG = Molecular Genetics/Cytogenetics/Genomics; MP = Molecular Pathology.

## Data Availability

The original contributions presented in this study are included in the article. Further inquiries can be directed to the corresponding author.

## Supplementary Materials

The following supporting information can be downloaded at https://www.mdpi.com/article/10.3390/curroncol33090505/s1, File S1: Charting the Future of Canadian Adult AML Laboratory Testing (phase I): Current State Mapping of Diagnostic AML Laboratory Practice.
