# Supplementary material for: Charting the Future of Canadian Adult Acute Myeloid Leukemia (AML) Laboratory Testing: A Canadian Leukemia Study Group Current State Mapping of Diagnostic AML Laboratory Practice
Source: Curr Oncol. 2026 Aug 25;33(9):505. doi: 10.3390/curroncol33090505 (PMC13605815; doi:10.3390/curroncol33090505)
Supplement: Supplementary file 1 [file curroncol-33-00505-s001.zip › curroncol-4454839-supplementary.pdf]

Charting the Future of Canadian Adult Acute Myeloid Leukemia (AML) Laboratory Testing: A Canadian Leukemia Study Group Current State Mapping of Diagnostic AML Laboratory Practice

Dear Laboratory Medicine Expert,

On behalf of the survey leads and the Canadian Leukemia Study Group (CSLG) Board, thank you for your interest in this mapping exercise. Your contributions will help to define current diagnostic testing practices across Canada for adult Acute Myeloid Leukemia (AML), including details pertaining to rapid testing, cytogenetics and next generation sequencing (NGS). Our aims are: 1) capture the current laboratory state; 2) identify barriers for future test development and; 3) forge a CLSG-supported network of laboratory medicine specialists with the common goal of advancing Canadian laboratory services in acute/chronic leukemias.

This phase I survey will focus on genomic test offerings in adult AML at diagnosis including methodology and target turn around time (TAT) to understand the variation in practices across the country. We hope this will form the basis of further engagement on challenges related to measurable residual disease (MRD), clinical deployment of optical genome mapping (OGM), and other topics pertinent to the laboratory aspects of acute leukemia care.

We are pleased to have participating stakeholders in British Columbia, Alberta, Saskatchewan, Manitoba, Ontario, Quebec and Nova Scotia (representing Atlantic Canada) in order to provide the most complete data set for potential development of national guidelines/recommendations.

As such, your participation is key to providing perspectives from your respective region.

Estimated Completion Time: 20-25 minutes

Questions:
hubert.tsui@sunnybrook.ca

Participant Information

1. Email:

   [ ]

2. Participant Name:

   [ ]

3. Please describe your professional background (check all that apply):
   - ☐ PhD
   - ☐ MD - Hematological Pathology
   - ☐ MD - Anatomic Pathology (Diagnostic and Molecular Pathology)
   - ☐ MD - General Pathology (Diagnostic and Clinical Pathology)
   - ☐ Fellowship Training in Molecular Genetics/Cytogenetics/Genomics (e.g. FACMG, FCCMG)
   - ☐ Fellowship Training in Molecular Pathology
   - ☐ Other: ____________________

4. Province:

   [ ]

5. Role:

   [ ]

6. Name of Institution:

   [ ]

Institutional Practice

7. Please describe the geographical catchment/regional coverage of your AML diagnostic testing (e.g. all of province, southern Alberta)

   [ ]

8. For any given individual AML patient, the following AML Diagnostic Services are performed *locally* at your centre (check all that apply):
   - ☐ Morphologic interpretation of aspirate/biopsy
   - ☐ Flow cytometry
   - ☐ Cytogenetics
   - ☐ Molecular
   - ☐ Other: ____________________

9. For AML, a single integrated report incorporating morphology, flow cytometry, cytogenetics and molecular is issued once results are finalized
   - ☐ Yes
   - ☐ No
   - ☐ Other: ____________________

10. To the best of your knowledge, a final classification (wherever possible) is issued for AML using the following classification system (check all that apply):
    - ☐ WHO 4th ed. revised (2016)
    - ☐ WHO 5th ed. (2022)
    - ☐ 2022 International Consensus Classification (ICC)
    - ☐ All three classifications are provided
    - ☐ Practice varies at our institution according to the pathologist
    - ☐ Other: ____________________

Laboratory Capabilities - AML Rapid Tests (rapid = 5 calendar day target TAT)

11. Do you offer in-house molecular rapid tests for the following genes/variants? (rapid = 5 calendar day target TAT)

	Yes	No	Referred Out
FLT3-ITD	☐	☐	☐
FLT3-TKD	☐	☐	☐
NPM1	☐	☐	☐
IDH1	☐	☐	☐
IDH2	☐	☐	☐
TP53	☐	☐	☐

12. Where applicable, what methodology do you use for the following molecular AML rapid tests? (rapid = 5 calendar day target TAT; please choose all that apply)
PCR: Polymerase chain reaction
qPCR: quantitative PCR
RT-PCR: Reverse transcription PCR
RT-qPCR: Reverse transcription quantitative PCR
ddPCR: digital droplet PCR
NGS: Next Generation Sequencing

	PCR	qPCR	RT-PCR	RT-qPCR	ddPCR	NGS
FLT3-ITD	☐	☐	☐	☐	☐	☐
FLT3-TKD	☐	☐	☐	☐	☐	☐
NPM1	☐	☐	☐	☐	☐	☐
IDH1	☐	☐	☐	☐	☐	☐
IDH2	☐	☐	☐	☐	☐	☐
TP53	☐	☐	☐	☐	☐	☐

13. If applicable, please provide any additional context or comments regarding the methodologies you use for the molecular AML rapid tests mentioned above. Specifically, please elaborate on why and when you use the different methodologies (optional):

14. Do you offer in-house molecular rapid tests for the following fusions? (rapid = 5 calendar day target TAT)

	Yes	No
BCR::ABL1	☐	☐
CBFB::MYH11	☐	☐
KMT2A rearrangements	☐	☐
PML::RARA	☐	☐
RUNX1::RUNX1T1	☐	☐

15. **Where applicable, what methodology do you use for rapid fusion detection in AML? (rapid = 5 calendar day target TAT; please choose all that apply)**
FISH: Fluorescent in-situ hybridization
OGM: Optical Genome Mapping

	PCR	RT-PCR	qPCR	RT-qPCR	Karyotype	FISH	OGM	NGS (e.g. fusion panel)	Not performed	Other
BCR::ABL1	☐	☐	☐	☐	☐	☐	☐	☐	☐	☐
CBFB::MYH11	☐	☐	☐	☐	☐	☐	☐	☐	☐	☐
KMT2A rearrangements	☐	☐	☐	☐	☐	☐	☐	☐	☐	☐
PML::RARA	☐	☐	☐	☐	☐	☐	☐	☐	☐	☐
RUNX1::RUNX1T1	☐	☐	☐	☐	☐	☐	☐	☐	☐	☐

16. If applicable, please provide any additional context or comments regarding the methodologies you use for rapid fusion detection in AML as mentioned above. Specifically, please elaborate on why and when you use the different methodologies (optional):

17. Where applicable, please describe the scenario for which rapid AML fusion tests are activated at your centre: (rapid = 5 calendar day target TAT)

	Activated only on clinical/laboratory suspicion (e.g. hematopathologist ordered)	Performed always when testing indication is suspected AML	Performed as requested on requisition, regardless of indication	Not performed	Other
BCR::ABL1	☐	☐	☐	☐	☐
CBFB::MYH11	☐	☐	☐	☐	☐
KMT2A rearrangements	☐	☐	☐	☐	☐
PML::RARA	☐	☐	☐	☐	☐
RUNX1::RUNX1T1	☐	☐	☐	☐	☐

18. If applicable, please provide any additional context or comments regarding the scenario for which rapid AML fusion tests are activated at your centre mentioned above (optional):

Rapid test methodology: FLT3-ITD

19. For rapid detection of diagnostic levels of FLT3-ITD do you use a commercial assay or internal laboratory developed test? (rapid = 5 calendar day target TAT)
☐Commercial Assay
☐Internal Laboratory Developed Test
☐Referred out
☐Not performed

20. If FLT3-ITD is tested with a commercial assay, what is the vendor and name of the kit? What is the analyte used (i.e. RNA, DNA)?

21. If FLT3-ITD testing is referred out, please list the external laboratory (Institution, Laboratory or Commercial Vendor):

22. What is your target TAT for diagnostic FLT3-ITD? (Please enter calendar days)

Rapid test methodology: FLT3-TKD

23. For rapid detection of diagnostic levels of FLT3-TKD do you use a commercial assay or internal laboratory developed test? (rapid = 5 calendar day target TAT)
☐Commercial Assay
☐Internal Laboratory Developed Test
☐Referred out
☐Not performed

24. If FLT3- TKD is tested with a commercial assay, what is the vendor and name of the kit? What is the analyte used (i.e. RNA, DNA)?

25. If FLT3-TKD testing is referred out, please list the external laboratory (Institution, Laboratory or Commercial Vendor):

26. What is your target TAT for diagnostic FLT3- TKD? (Please enter calendar days)

Rapid test methodology: NPM1

27. For rapid detection of diagnostic levels of NPM1 do you use a commercial assay or internal laboratory developed test? (rapid = 5 calendar day target TAT)
    - ☐ Commercial Assay
    - ☐ Internal Laboratory Developed Test
    - ☐ Referred out
    - ☐ Not performed

28. If NPM1 is tested with a commercial assay, what is the vendor and name of the kit? What is the analyte used (i.e. RNA, DNA)?

29. If NPM1 testing is referred out, please list the external laboratory (Institution, Laboratory or Commercial Vendor):

30. What is your target TAT for diagnostic NPM1? (Please enter calendar days)

Rapid test methodology: IDH1

31. For rapid detection of diagnostic levels of IDH1 do you use a commercial assay or internal laboratory developed test? (rapid = 5 calendar day target TAT)
    - ☐ Commercial Assay
    - ☐ Internal Laboratory Developed Test
    - ☐ Referred out
    - ☐ Not performed

32. If IDH1 is tested with a commercial assay, what is the vendor and name of the kit? What is the analyte used (i.e. RNA, DNA)?

33. If IDH1 testing is referred out, please list the external laboratory (Institution, Laboratory or Commercial Vendor):

34. What is your target TAT for diagnostic IDH1? (Please enter calendar days)

Rapid test methodology: IDH2

35. For rapid detection of diagnostic levels of IDH1 do you use a commercial assay or internal laboratory developed test? (rapid = 5 calendar day target TAT)
    - ☐ Commercial Assay
    - ☐ Internal Laboratory Developed Test
    - ☐ Referred out
    - ☐ Not performed

36. If IDH2 is tested with a commercial assay, what is the vendor and name of the kit? What is the analyte used (i.e. RNA, DNA)?

37. If IDH2 testing is referred out, please list the external laboratory (Institution, Laboratory or Commercial Vendor):

38. What is your target TAT for diagnostic IDH2? (Please enter calendar days)

AML Next Generation Sequencing (NGS)

39. What myeloid NGS platform do you deploy at diagnosis of AML?
    - ☐ Commercial Assay
    - ☐ Internal Laboratory Developed Test
    - ☐ Referred out
    - ☐ Not performed

40. If your myeloid NGS testing is a commercial panel, please list the kit used and the commercial vendor. Please also indicate the analyte used (i.e. RNA, DNA)?

41. If myeloid NGS testing is referred out, please list the external laboratory (Institution, Laboratory or Commercial Vendor):

42. What is your target TAT for AML myeloid NGS? (Please enter calendar days)

43. Is the NGS panel used for AML the same as that used for myelodysplastic syndrome (MDS), myeloproliferative neoplasms (MPN) and myelodysplastic/myeloproliferative neoplasms (MDS/MPN)?
    - ☐ Yes
    - ☐ No
    - ☐ Other: ______________________________

AML Cytogenetics: The following section refers to cytogenetic testing outside of rapid fusion detection by FISH

44. What is your first-line cytogenetic methodology used for diagnostic cases of AML?
    - ☐ Karyotype
    - ☐ Optical Genome Mapping (OGM)
    - ☐ FISH
    - ☐ Microarray
    - ☐ Other: ______________________________

45. What is your target TAT for first-line AML cytogenetics? (Please enter calendar days)

46. If first-line cytogenetic methodology fails or is suboptimal, which second-line reflex testing methods do you use? (check all that apply):
☐ Karyotype
☐ Optical Genome Mapping (OGM)
☐ FISH
☐ Microarray
☐ Not applicable

47. When FISH is reflexed for AML, is there a standard FISH panel that is always used when cytogenetic analysis fails, or in cases such as suspicion of a low-level clone with +8 or rearrangements, etc.?
☐ Yes
☐ No
☐ Other: ____

48. If you answered "yes" to the question above, please elaborate on which probes are used in the comments section here (please indicate N/A if you answered "No" and proceed to the next question):

[ ]

49. If a standard FISH panel is not routinely used, which FISH probes are available for testing at your institution?

[ ]

50. When FISH is performed for AML, which of the following chromosomes/genes are evaluated? (check all that apply):
☐ Chr 5
☐ Chr 7
☐ Chr 8
☐ Chr 12
☐ Chr 17
☐ Chr 20
☐ KMT2A
☐ MECOM
☐ RUNX1::RUNX1T1
☐ CBFB::MYH11
☐ PML::RARA
☐ Other: ____

51. The category of myeloid/lymphoid neoplasms with eosinophilia and tyrosine kinase gene mutations may present as acute leukemia including AML. Please describe how the following are assessed at your centre:

	Karyotype	OGM	FISH	Single gene molecular methods	NGS	Not performed	Other
PDGFRA rearrangement	☐	☐	☐	☐	☐	☐	☐
PDGFRB rearrangement	☐	☐	☐	☐	☐	☐	☐

FGFR1 rearrangement	☐	☐	☐	☐	☐	☐	☐
JAK2 rearrangement	☐	☐	☐	☐	☐	☐	☐
FLT3 rearrangement	☐	☐	☐	☐	☐	☐	☐
ETV6::ABL1	☐	☐	☐	☐	☐	☐	☐

52. If applicable, please provide any additional context or comments regarding the question above (optional):

53. Some recurrent AML fusions are known to be difficult to detect by conventional karyotyping (i.e. PDGFRA rearrangement, etc.). Your current workflow for cryptic rearrangements involves (check all that apply):

☐Targeted testing such a FISH but only when specifically indicated (e.g. eosinophilia is present)
☐Targeted testing for all suspected AML cases
☐Rely on OGM for detecting cryptic fusions
☐Rely on NGS for detecting cryptic fusions
☐Other: ____

54. If applicable, please provide any additional context or comments regarding the question above (optional):

Turn Around Time: In 2022, the European LeukemiaNet (ELN) published updated recommendations on turn around times for diagnostic AML genomic results (PMID 35797463).

55. For your jurisdiction, AML genomic TAT targets are determined by (check all that apply):

☐Internal consensus at the institution (i.e. hematologists and clinical /operational laboratory staff)
☐Stated formally at the provincial level (e.g. provincial cancer agency)
☐External institution / laboratory
☐International recommendations/guidelines (e.g. ELN, NCI)
☐Other: ____

56. The 2022 ELN AML Guidelines indicate a preferred TAT for AML rapid tests of 3-5 calendar days for FLT3, NPM1, IDH1 and IDH2. For your current suite of rapid tests, please describe TAT relative to 2022 ELN recommendations:

	Our current TAT targets are typically concordant with 2022 ELN	Our current TAT targets are typically longer than 2022 ELN	Not performed/resulted as a rapid test
FLT3-ITD	☐	☐	☐
FLT3-TKD	☐	☐	☐
NPM1	☐	☐	☐
IDH1	☐	☐	☐
IDH2	☐	☐	☐

57. The 2022 ELN AML Guidelines indicate a preferred TAT for additional AML genes within the first cycle of treatment (e.g. 21 calendar days). For AML myeloid NGS, please describe your TAT relative to 2022 ELN recommendations:

☐Our current TAT targets are typically concordant with 2022 ELN
☐Our current TAT targets are typically longer than 2022 ELN
☐Other

58. Where either ELN TAT and/or current institutional/provincial TAT targets are ongoing challenges, what are the priority areas for future investment (check all that apply):
☐Human health resources
☐Infrastructure (e.g. new technologies, bioinformatics, etc)
☐Utilization recommendations (e.g. CLSG guidelines)
☐A need for more coordinated, centralized testing (e.g. regional/provincial)
☐Improved alignment between clinically targetable/actionable biomarkers and laboratory TAT
☐Other: ____

Conceptualizing Future National Collaboration: Thank you for your time and input thus far. The last few questions are intended to direct the discussion for future engagement and a phase II survey.

59. What are your priority projects in advancing AML laboratory diagnostics (check all that apply)?

☐Validation of single biomarker commercial AML MRD assays (e.g FLT3-ITD)
☐Validation of single biomarker in-house AML MRD assays (e.g FLT3-ITD)
☐Validation of multiple biomarker, NGS-based commercial AML MRD assays
☐Validation of multiple biomarker, NGS-based in-house AML MRD assays
☐Clinical deployment of Optical Genome Mapping (OGM)
☐Reduce the TAT of diagnostic myeloid NGS
☐Reduce the TAT of diagnostic AML cytogenetic results
☐AML Whole Genome Sequencing
☐AML RNA sequencing
☐AML Single cell sequencing
☐Germline testing for all or selected AML patients at diagnosis (e.g. from skin fibroblasts)
☐Other: ____

60. Optional: What other new technologies or methodologies are you considering for implementation in your laboratory for myeloid neoplasms?

61. Do you agree that Canadian guidelines / recommendations (e.g. led by laboratory specialists as part of the CLSG) in AML diagnostic testing would help to support further laboratory investment and advocacy?

☐ Strongly Disagree	☐ Disagree	☐ Neutral	☐ Agree	☐ Strongly Agree

62. Would you be interested in joint (national) validation projects in AML diagnostic testing?
☐ Yes
☐ No
☐ Maybe
☐ Other: ____________________

63. Would you be open to pharmaceutical companies sponsoring joint validation projects in AML?
☐ Yes
☐ No
☐ Maybe
☐ Other: ____________________

64. Do you have any additional comments or suggestions regarding AML diagnostics in Canada?

